# Improving the safety of oral immunotherapy for food allergy

**DOI:** 10.1111/pai.12510

**Published:** 2015-12-22

**Authors:** Marta Vazquez‐Ortiz, Paul J. Turner

**Affiliations:** ^1^ Section of PaediatricsImperial College LondonLondonUK; ^2^ Discipline of Paediatrics and Child HealthSchool of MedicineUniversity of SydneySydneyNSWAustralia

**Keywords:** children, food allergy, oral immunotherapy, quality of life, safety

## Abstract

Food allergy is a major public health problem in children, impacting upon the affected individual, their families and others charged with their care, for example educational establishments, and the food industry. In contrast to most other paediatric diseases, there is no established cure: current management is based upon dietary avoidance and the provision of rescue medication in the event of accidental reactions, which are common. This strategy has significant limitations and impacts adversely on health‐related quality of life. In the last decade, research into disease‐modifying treatments for food allergy has emerged, predominantly for peanut, egg and cow's milk. Most studies have used the oral route (oral immunotherapy, OIT), in which increasing amounts of allergen are given over weeks–months. OIT has proven effective to induce immune modulation and ‘desensitization’ – that is, an increase in the amount of food allergen that can be consumed, so long as regular (typically daily) doses are continued. However, its ability to induce permanent tolerance once ongoing exposure has stopped seems limited. Additionally, the short‐ and long‐term safety of OIT is often poorly reported, raising concerns about its implementation in routine practice. Most patients experience allergic reactions and, although generally mild, severe reactions have occurred. Long‐term adherence is unclear, which rises concerns given the low rates of long‐term tolerance induction. Current research focuses on improving current limitations, especially safety. Strategies include alternative routes (sublingual, epicutaneous), modified hypoallergenic products and adjuvants (anti‐IgE, pre‐/probiotics). Biomarkers of safe/successful OIT are also under investigation.

Food allergy is a major public health issue throughout the world, particularly in children. There is no established treatment for use in routine clinical practice: management involves avoidance of the culprit food(s) and rescue medication in the event of accidental reactions 1. Food allergy impacts significantly on health‐related quality of life (HRQoL) of both the affected individual and their families. The last decade has seen an increase in research into possible treatments for food allergy.

## Impact of food allergy

Food allergy is estimated to affect up to 6% of children in Europe 2. The incidence is rising, with an 18% increase in children in the last decade in the USA 3. Hospital admissions due to anaphylaxis – the most severe manifestation of food allergy – have doubled in the UK from 1.2 per 100,000 population per annum in 1992 to 2.4 in 2012 4, especially in children (0–14 years).

Cow's milk, egg, peanut and tree nuts are the most common food allergens in children 2. Variations in the prevalence of allergy to different foods between countries may depend on local dietary preferences 5. Whether local consumption patterns also affect resolution of food allergies is unknown. Peanut allergy persists into adulthood in 80% of cases 6, 7. In contrast, around 50% of children with cow's milk and/or egg allergy develop tolerance within the first 5–6 years of life 8, 9. Persistence of reactivity to the latter two allergens is a significant concern, because individuals frequently have more severe and complex allergic phenotype 8, 9. Given the high incidence of milk and egg allergy in the general population, the absolute numbers of those with persistent disease are significant, particularly in tertiary care 10, 11.

The spectrum of severity for symptoms during food‐allergic reactions is variable and includes life‐threatening anaphylaxis and even death 12. Cow's milk and peanuts were the most common triggers for fatal anaphylaxis in UK children between 1992 and 2012 4. Fortunately, fatal anaphylaxis, while unpredictable, is also very uncommon, with an incidence rate of 1.81 per million person‐years (95% CI 0.94–3.45) 12. However, our inability to predict those most at risk of severe reactions contributes to the widespread provision of rescue medication (such as adrenaline auto‐injectors) and anxiety which impacts adversely on HRQoL to a greater degree than that reported for chronic illnesses such as diabetes or idiopathic arthritis 13, 14. The most common childhood allergens – especially cow's milk – are key dietary constituents providing essential nutrients needed for growth and development. Dietary elimination can therefore be challenging, especially in those with multiple food allergies 15. Finally, food allergy is a major public health issue, affecting the food industry with regard to allergen risk management and mandatory allergen labelling 16. There are further cost implications, not only for affected families but also to the health system, with a doubling of direct health costs compared to non‐allergic individuals 17.

## Limitations of current management for food allergy

Even with appropriate dietary avoidance, accidental allergic reactions are common: up to 40% of children allergic to cow's milk had at least one reaction every year according to one report 18. Strict avoidance in children is difficult, because the most common food allergens (cow's milk, egg or nuts) are present in many dishes and manufactured products. Moreover, allergen labelling can be poor and confusing, increasing the potential for inadvertent allergen consumption 19. This is a particular issue with precautionary allergen labelling: a UK‐based survey found 69% of cereals and 56% of confectionery items have ‘may contain’ labelling to nuts, despite nuts not being present as an ingredient 20; such labelling is often ignored by consumers 21.

Many factors contribute to rescue medication often not being used appropriately in the community when needed. Many parents/patients – especially teenagers – do not carry their medication at all times, find it difficult to identify anaphylaxis symptoms or to use auto‐injector effectively in these stressful situations 22, 23. Others are frightened because of needle‐phobia or possible side effects 24. Many caregivers do not receive any formal training on anaphylaxis management from healthcare professionals. All of these issues may affect the ability of staff within education establishments (e.g. schools) to correctly identify and administer emergency medication in the event of a reaction 25. Finally, fatal cases of food anaphylaxis have been reported, even when adrenaline was administered correctly in a timely manner 26, 27.

## Oral immunotherapy for food allergy

Given the above, there is demand for a disease‐modifying treatment for food allergy, particularly for the most common, ubiquitous and dangerous allergens in terms of fatal anaphylaxis: cow's milk and peanut. Different strategies are under investigation, the most common approach being oral immunotherapy (OIT) with over 60 studies published in peer‐review journals. Results have been promising in terms of effectiveness and positive impact on parent‐assessed HRQoL. However, significant concerns remain regarding the safety of OIT, and its ability to induce permanent tolerance. Current research focuses in improving these two key issues. The benefits and pitfalls of OIT are summarized in Table 1.

**Table 1:** Benefits and pitfalls of oral immunotherapy for food allergy

Benefits	Pitfalls
Ability to induce desensitization	Limited ability to induce permanent tolerance
Improvement in Quality of life: Dietary limitations Social restrictions Emotional impact Anxiety Improved nutrition	Increased risk for allergic reactions Need for regular long‐term consumption Need for extensive monitoring, including long term (resource‐consuming) Failure rate: around 10–35% due to significant allergic reactions
Potential protection against accidental reactions	Relatively high long‐term dropout rate

### Rationale and underlying immune modulation

OIT consists of giving increasing amounts of food allergen orally over weeks or months (‘updosing phase’). Once the target dose is reached, this amount is given on a regular basis, usually daily (‘maintenance phase’). This results in an allergen‐specific immunomodulatory effect. A recent meta‐analysis showed a reduction in skin prick test wheal size and an increase in specific IgG4‐blocking antibodies following OIT to cow's milk, egg and peanut 28, with the latter possibly being a biomarker for sustained unresponsiveness 29. A trend towards a reduction in specific IgE levels was also detected. The underlying immune mechanisms are not fully understood. Exposure to low allergen doses seem to promote inducible T‐regulatory cells (CD25+ FoxP3+) in gut MALT tissue, which reduces allergen‐specific Th2 response through IL10 and TGFβ 30. Exposure to high allergen doses might induce allergen‐specific T‐cell anergy or clonal deletion 31.

### Effectiveness

OIT is effective in increasing the amount (or threshold) of allergen food‐allergic individuals are able to eat without experiencing an allergic reaction, an effect termed desensitization. A meta‐analysis by Nurmatov et al. 28 reported a significant reduction in the likelihood of reacting at a food challenge following OIT compared to controls (Risk ratio: 0.21, 95% CI: 0.12–0.38). However, the ability of OIT to induce permanent or sustained tolerance – once ongoing exposure has stopped – has not, to date, been extensively evaluated. The available published data are not encouraging. In a randomized controlled trial (RCT) of OIT for egg and cow's milk allergy in 45 young children, tolerance rates were equal (35%) in both the OIT and control group after OIT (median duration 21 months) followed by 2 months off‐OIT 32. In a RCT of egg OIT, 75% (30/40) of children were desensitized at 22 months, but only 27.5% (11/40) were tolerant after stopping OIT for 4–6 weeks 33. Similar findings have been reported for peanut OIT: one uncontrolled study found that only 50% (12/24) of patients who had reached the target peanut dose (4000 mg) maintain tolerance 4 weeks after stopping OIT 34.

It seems a little incongruous that for other forms of immunotherapy – such as subcutaneous immunotherapy (SCIT) for aeroallergens and venom, success is generally defined as tolerance (and *not* transient desensitization) after 3–5 years of active treatment – yet the same criteria are not applied to OIT. Further research is needed to clarify whether a longer course of OIT would increase rates of tolerance, and whether OIT only accelerates allergy resolution in those who would have developed natural tolerance without any intervention. As it is difficult to determine for how long OIT maintenance should be stopped to prove permanent tolerance (most studies utilize a ‘short’ off‐OIT phase of 2–8 weeks), the term ‘sustained unresponsiveness’ is preferred instead of tolerance 33.

### Safety

The main limitation to OIT, and arguably the reason why the international consensus is that it is not yet ready for routine clinical practice, is the risk of allergic reactions 35. The lack of consensus in safety reporting (in contrast to other forms of allergen immunotherapy) makes it extremely difficult to appraise the frequency of reactions and thus the safety of OIT 30. Many studies focus on effectiveness and provide limited safety data. The meta‐analysis by Nurmatov et al. 28 could identify data relating to systemic adverse reactions in only 5 of the 21 studies included. An increased risk for local reactions with OIT was reported (RR: 1.47; 95% CI: 1.11–1.95), with a similar trend for systemic reactions, especially for cow's milk OIT 30. A previous meta‐analysis assessing cow's milk OIT reported an increased risk with OIT for adrenaline use (RR: 5.8; 95% CI: 1.6–21.9) and for all types of allergic reactions, including those potentially life‐threatening such as bronchoconstriction (RR: 10; 95% CI: 2.4–41.4) or laryngospasm (RR: 12.9; 95% CI: 1.7–18.6) 36.

Although these results are self‐explanatory, they do not fully show the complexity of OIT safety in terms of the frequency of reactions, their severity, evolution over time and potential relationship with cofactors or poor adherence to the OIT protocol. These essential safety data are lacking in most studies on OIT. Safety data may be provided as the proportion of doses causing reactions, rather than the proportion of patients experiencing a reaction. For example, if 5 individuals (in study of 50 patients undergoing daily OIT over 6 months) experience anaphylaxis to an OIT home dose, this at could be reported as a rate of 10% (patients) or 0.05% of doses (9125 doses administered over the 6 months). Furthermore, different classification systems are used to describe severity across studies, and the indications to administer adrenaline can vary considerably. In this context, there are major limitations to compare safety outcomes across studies and identify optimal OIT regimens.

On the one hand, most studies report that OIT‐related reactions are generally mild and usually tend to decrease or resolve overtime. Probably many patients may undergo successful OIT without major safety concerns 37, 38, 39. On the other hand, worrying safety data are reported in many studies: reactions occur in most patients. Studies report that 10–35% of children need to be withdrawn due to significant and/or repeated reactions; cough, wheeze or stridor (which can be regarded as potentially life‐threatening) are not uncommon 40, 41, 42. Near‐fatal reactions have been reported in asthmatic teenagers with poor compliance 43, 44. This raises concerns about long‐term safety, especially when reaching adolescence, if permanent tolerance is not achieved. Importantly, poor long‐term adherence has been reported in over 60% of cases after 3–5 years on cow's milk and peanut OIT 45, 46.

### Impact on quality of life, nutrition and health economics

A few open studies of OIT have shown a positive impact on children's quality of life following successful OIT using validated questionnaires to assess HRQoL 42, 47, 48, 49; however, in almost all cases, these assessments have been made in the parents and not in the children themselves undergoing OIT, despite such questionnaires being available for use in children from age 8 years. Given the potential for participation in OIT being influenced considerably by parents (rather than being up to the child), it is important that the child's perspective and views on OIT are explored. No studies have addressed the impact of OIT on HRQoL in the longer term, or in those patients who fail OIT due to reactions. A recent study reported that parents only perceived a very limited benefit from the child undergoing egg OIT; interestingly, the improvement in HRQoL correlated inversely with the frequency of OIT‐related reactions, suggesting that if safety is compromised, quality of life does not improve 50.

No studies have, as yet, assessed whether successful OIT improves nutrition by allowing the child to re‐introduce foods (such as cow's milk) into their diet, although the impact on nutrition may be limited given that OIT is not generally performed in very young children. Studies on cost‐effectiveness of OIT are also lacking at this time. OIT studies to date have focused on efficacy as the primary outcome. Whether this is the most important outcome for patients has not been evaluated. There is a need to involve patients and caregivers in defining relevant outcomes for studies on treatments for food allergy.

## Strategies to improve the safety of immunotherapy for food allergy

**Figure 1:**
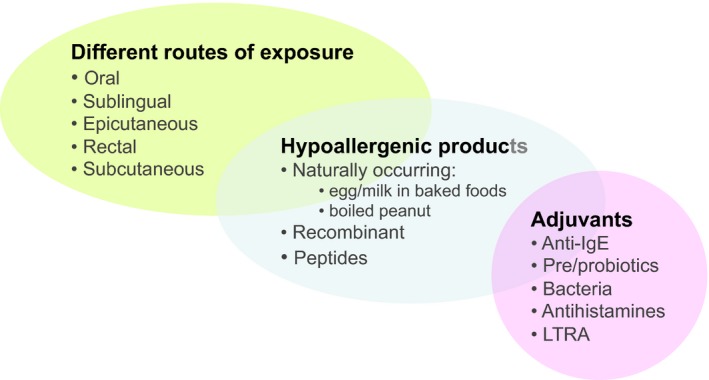
Strategies under investigation to improve the efficacy and safety of OIT. LRTA, leukotriene receptor antagonists.

Several approaches have been proposed to improve the safety of OIT. These include research on using alternative routes of exposure, modified hypoallergenic products and adjuvants for immunotherapy as well as on biomarkers of safe and successful OIT to facilitate patient selection (Fig. 1).

### Biomarkers of safe and successful OIT

The identification of biomarkers associated with safe (and successful) OIT might help select suitable candidates more likely to respond safely to OIT, and screen out those candidates in whom OIT may lead to unnecessary risks. Although the outcome of OIT probably depends on multiple factors, some individual characteristics might have a predictive value. In a recent study of cow's milk OIT, patients who were withdrawn due to significant reactions had IgE binding to a broader diversity of peptides (especially to alpha‐s1‐casein) and at higher intensity than those children who completed OIT successfully 29. In a further study, children with frequent and severe reactions to CM‐OIT had different baseline characteristics compared to those who tolerated OIT: higher CM‐specific IgE levels prior to OIT, more severe reactions to low CM doses and more severe asthma 41. The same factors were associated to early withdrawal on egg OIT in another study 51, suggesting that OIT might be unsafe in those individuals with persistent and more severe allergy to cow's milk and/or egg allergy. Excluding these ‘high‐risk’ children may seem reasonable, but it is these patients who have most to benefit from OIT.

### Alternative routes

#### Sublingual immunotherapy

Several studies have used the sublingual route for food immunotherapy (peanut, cow′s milk, hazelnut, peach). This approach builds on existing evidence on the efficacy and good safety profile of sublingual immunotherapy (SLIT) for inhalant allergens 52. Effector cells (mast cells, basophils) are scarce, in the sublingual mucosa which reduces the risk of allergic reactions. Conversely, antigen‐presenting dendritic cells (e.g. Langerhans cells) are abundant, which may promote the induction of tolerogenic T‐regulatory cells 53. SLIT for food allergy mimics some of the immune changes seen with OIT. SLIT for food allergy can result in decreased titrated skin prick test and specific IgE levels, with associated allergen‐specific increases in IgG4 46, 54, 55, 56. Systemic reactions are less frequent on SLIT, although they still may occur, especially if the allergen is ingested 54, 57. A recent meta‐analysis found SLIT was effective at inducing desensitization 28; however, the increase in amount of food allergen that can be tolerated following SLIT is modest and lower than that achievable with OIT 54, 55, 56, 57. Two studies have compared the efficacy of SLIT vs. OIT for cow′s milk and peanut allergy, respectively. In both cases, OIT resulted in an increase in threshold up to 10 times that achieved with SLIT 54, 57. Whether the effect of SLIT is sufficient to protect patients from accidental reactions is unclear, as the median threshold following SLIT can be relatively low: a study of peanut SLIT resulted in a median threshold for reactivity of 371 mg of peanut protein – less than 2 peanuts 58. Effectiveness appears to improve with more prolonged treatment for SLIT to cow's milk 57, but similar studies with peanut SLIT have provided conflicting results 58. The lower doses used for SLIT (in comparison with OIT) may contribute to its limited efficacy, but better safety profile. Whether the dose used for SLIT can be increased to try to improve efficacy without compromising safety requires investigation. Concentrating the SLIT extract to deliver higher doses did not improve efficacy in one study, although given the high dropout rate seen, these data must be interpreted with caution 46. Finally, a recent trial in peanut‐allergic children suggested that pre‐treatment with SLIT may improve the safety of subsequent OIT 54. This approach is deserving of further evaluation.

#### Epicutaneous immunotherapy

Epicutaneous immunotherapy (EPIT) involves the application of a patch containing the food allergen to the skin. The epidermis is poorly vascularized, which might limit the potential for systemic reactions. In contrast, it contains high numbers of potent antigen‐presenting cells, which may allow immune modulation and enhanced efficacy 59. Pre‐clinical data in peanut‐sensitized mice demonstrated that peanut‐EPIT on intact skin decreased the clinical and allergen‐specific Th2 responses, and increased local and peripheral Foxp3+ Tregs 60. Furthermore, Tregs persisted for 8 weeks after the end of EPIT, suggesting that EPIT might induce long‐term tolerance 61. A pilot study in 19 children with cow's milk allergy using EPIT for 3 months found a tendency towards an increased cumulative dose, but this was not statistically significant. No systemic reactions occurred; however, local eczematous skin reactions were common 62. Larger studies with longer duration are required to evaluate the effectiveness and safety of EPIT for food allergy in humans, and phase I‐II studies in peanut‐allergic patients are underway (Clinicaltrials.gov NCT01170286 and NCT01197053). A further possibility is that EPIT could be combined with other routes of immunotherapy (OIT or SLIT), to improve safety and efficacy or perhaps as an alternative route of providing ongoing maintenance therapy.

#### Subcutaneous immunotherapy

The first trial on immunotherapy for food allergy was a RCT performed in the 1990s in 12 peanut‐allergic adults using SCIT (aqueous peanut extract) for 1 year 63. All 6 patients on active treatment required intramuscular adrenaline for systemic reactions on more than one occasion, with anaphylaxis occurring during both the rush and maintenance phases. While SCIT resulted in an increase in threshold of reactivity at DBPCFC, the authors acknowledged that SCIT using native food allergen was unsafe. Subsequent studies have focused in the development of hypoallergenic products to improve the safety of SCIT.

### Modified hypoallergenic molecules

The underlying rationale is to use products that have reduced or no ability to bind specific IgE and activate mast cells and basophils through modification of allergenic sites or epitopes. However, their ability to interact with T cells is preserved to allow immune modulation without triggering allergic reactions. Such products can be found ‘naturally’ in our diets or produced in the laboratory.

#### Naturally occurring hypoallergenic foods

There is now strong evidence that baked foods containing extensively heated cow's milk or egg in baked foods (such as biscuits, cakes or muffins) are tolerated by approximately 70% of children with milk or egg allergy 64, 65, 66. This is a result of heat‐induced protein structure modification and effects from a gluten‐containing food matrix (such as the formation of protein aggregates), altering the ability of IgE to bind to mainly conformational epitopes (as opposed to linear epitopes, which are heat resistant) and cause effector cell activation 67, 68, 69. Introducing baked foods containing egg/milk into the diet of children who are otherwise allergic to the native allergen may be a safe and simple way of accelerating natural tolerance, with both clinical and laboratory data suggesting this is the case 70, 71. However, it is difficult to demonstrate that children who tolerate extensively heated allergen outgrow their allergy due to exposure to the allergen in baked foods, or whether these individuals would outgrow their allergy through natural resolution, *independent* of allergen consumption. Indeed, having IgE against specific linear peptides/epitopes is associated with clinical reactivity to baked foods, and a more persistent course of cow's milk and egg allergy 72, 73. This dilemma is difficult to test in research, due to both ethical and pragmatic reasons (a family who know their child tolerates the allergen in baked foods is unlikely to agree to strict allergen avoidance, especially when exposure may help ‘cure’ their child).

A novel approach to peanut desensitization is currently being tested in a Phase 2/3 study (NCT02149719) using boiled peanut. This has reduced allergenicity compared to raw or roasted peanut (the latter being the usual type of peanut consumed in western countries). Boiling appears to result in the loss of key allergenic components (especially Ara h 1, 2 and 6) into the cooking water 74, 75. Preliminary data suggest that boiled peanut OIT might be a safe and effective to induce desensitization in children with peanut allergy 75.

#### Engineered recombinant proteins

The introduction of point mutations into known IgE‐binding epitopes of food allergens, either through chemical modification or site‐directed mutagenesis, can be used to develop recombinant proteins with reduced allergenicity. Hypoallergenic mutants of peanut, fish and apple allergens have been generated using the latter technique 76. Wood et al. recently published a phase 1 trial of a rectally administered suspension of recombinant dominant peanut allergens (Ara h 1, Ara h 2 and Ara h 3), modified by amino acid substitutions at major IgE‐binding epitopes and encapsulated in heat/phenol killed *E. coli* (EMP‐123). Unfortunately, 5 of 10 peanut‐allergic adults required discontinuation due to significant allergic reactions 77. It is not clear as to whether these outcomes were affected by the route of exposure. An ongoing EU‐funded study aims to develop hypoallergenic recombinant major allergens of fish (parvalbumin) and peach (lipid transfer protein) to be used as active ingredients of SCIT for food allergy 78.

#### Peptides

This approach uses overlapping peptides (protein fragments 10–20 amino acids long) which represent the entire sequence of the allergenic protein. These short peptides cannot cross‐link two IgE molecules, but can interact with antigen‐presenting cells. Such approaches have been successfully used for immunotherapy to aeroallergens 79. A peptide mixture of Ara h 2, the major peanut allergen, has been tested in a mouse model of peanut allergy with promising clinical and immunological results 80. The most relevant tolerogenic peptides need to be identified before human studies can be considered 81.

### Adjuvants

#### Anti‐IgE therapy

Anti‐IgE appears to be is a very promising therapy for IgE‐mediated allergic diseases, and probably acts by two mechanisms: preventing free IgE molecule from binding to its receptors on effector cells, and through effects on effector cells including by downregulating the expression of the high‐affinity IgE receptor on mast cells, and decreasing basophil histamine release 82. Evidence supporting its use in the management of food allergy is encouraging but currently limited, as reviewed elsewhere 83. In a double‐blind placebo‐controlled RCT in 84 peanut‐allergic adults, four doses of anti‐IgE were given at 4 weekly intervals (given its half‐life of 26 days) 84. An increase in the median threshold of reactivity from half a peanut before treatment to 9 peanuts was observed, without the use of a desensitization protocol. However, 25% of patients did not respond, and no long‐term data are described. Some studies have demonstrated the great potential of anti‐IgE in combination with OIT to allow both more rapid desensitization and improved safety. Two pilot studies in children allergic to cow's milk (n = 11) and peanut (n = 13) have been published, using anti‐IgE from at least 8 weeks prior to OIT commencement 85, 86. Around 80% of participants reached the target dose after 7–11 weeks of updosing (2000 mg cow's milk and 4000 mg peanut protein), an effect which persisted allowing tolerance to higher doses (8000 mg) at DBPCFC 8–12 weeks after stopping anti‐IgE. Most patients experienced only mild allergic reactions; 2 children on peanut OIT experienced bronchial reactions 86. During cow's milk OIT, no bronchial, laryngeal or cardiovascular reactions occurred (although 4 patients were given adrenaline nonetheless) 85. A more recent study performed simultaneous OIT to multiple foods using anti‐IgE from 8 weeks prior to 8 weeks after starting OIT 87. All children had reacted to doses <100 mg protein prior to OIT, and all tolerated 4000 mg for each allergen by 9 months of OIT (median time: 18 weeks). We are also aware of anecdotal reports where anti‐IgE has successfully been used as an adjuvant in desensitization in individuals with previous multiple episodes of anaphylaxis to LTP. Although anti‐IgE shows great promise in combination with OIT, more research is needed to address unclear issues before it should be considered ready for use outside the research setting. First, longer‐term effectiveness of OIT once anti‐IgE is stopped requires further investigation: there are case reports of IgE‐facilitated OIT where clinical symptoms to the allergen in question recurred following withdrawal of anti‐IgE therapy 88. Anti‐IgE does not seem to be equally effective in all patients (and this is directly related to OIT safety); more research is needed into understanding the reason for this, as well as into potential biomarkers to predict individual treatment responses. Finally, the high cost of anti‐IgE therapy is likely to be prohibitive in many countries.

#### Pre‐/probiotics

Evidence from animal models and *in vitro* studies suggest that gut microbiota modulate immune programming, promote oral tolerance and are important inhibiting the development of the allergic phenotype. The utility of pre‐biotics (non‐digestible carbohydrates that stimulate the growth and/or activity of beneficial colonic bacteria), probiotics (live microorganisms that benefit the host) and/or symbiotics (a combination of both) in the prevention and/or treatment of allergic diseases has been addressed in different studies 89. Results from animal models are encouraging, but in‐human data are minimal. A recent study addressed the potential of probiotics to induce beneficial gut immune modulation in association with peanut OIT 90. Tolerance rates after stopping OIT were higher than in previous studies. However, definitive conclusions cannot be drawn, because the authors did not include a control group receiving OIT without probiotics. Around half of the patients experienced significant allergic reactions to OIT.

#### Bacteria

As bacteria are potent stimulants of Th1 immune responses, modified bacterial products are under investigation as adjuvants for allergen‐specific immunotherapy. In a dog model of food allergy, a single subcutaneous injection with a mixture of heat‐killed Listeria monocytogenes (HKLM) and either peanut, or milk and wheat, significantly reduced anaphylactic symptoms on oral food challenge 91. Heat/phenol killed *E. coli* was associated to the above‐mentioned rectal EMP‐123 vaccine for peanut allergy 77.

#### Antihistamines and leukotriene receptor antagonists

Daily antihistamines have been used during OIT updosing in several studies 37, 40, 92. This is expected to reduce mild oral, skin, nasal and/or ocular symptoms related to OIT doses. There is anecdotal evidence of the potential usefulness of leukotriene receptor antagonists to treat gastrointestinal symptoms during OIT 93. However, the impact on these treatments on OIT safety cannot be determined, as no RCTs have been performed.

## Long‐term safety

There is little data relating to the longer‐term safety of OIT. Clearly, a major issue is that of sustained unresponsiveness vs. transient desensitization, the latter requiring ongoing regular exposure to maintain desensitization. One concern remains issues of compliance, particularly in teenagers where this can be a problem during OIT 43. It is not uncommon for individuals who have undergone OIT to continue to experience aversion and/or oral symptoms to maintenance doses. It is not difficult to foresee a scenario where an individual is not compliant with their maintenance dosing, but believes they are now ‘tolerant’ to the allergen in question – thus risking the possibility of a severe reaction in the event of future allergen exposure.

Finally, there is also a concern that OIT can result in the development of eosinophilic oesophagitis (EoE) and other food‐induced enteropathies. A systematic review reported EoE in up to 2.7% of patients undergoing OIT for IgE‐mediated food allergy (although the review is based on incomplete datasets, because most trials of OIT have not reported the presence or absence of EoE as a longer‐term adverse event) 94.

## Conclusions

**Figure 2:**
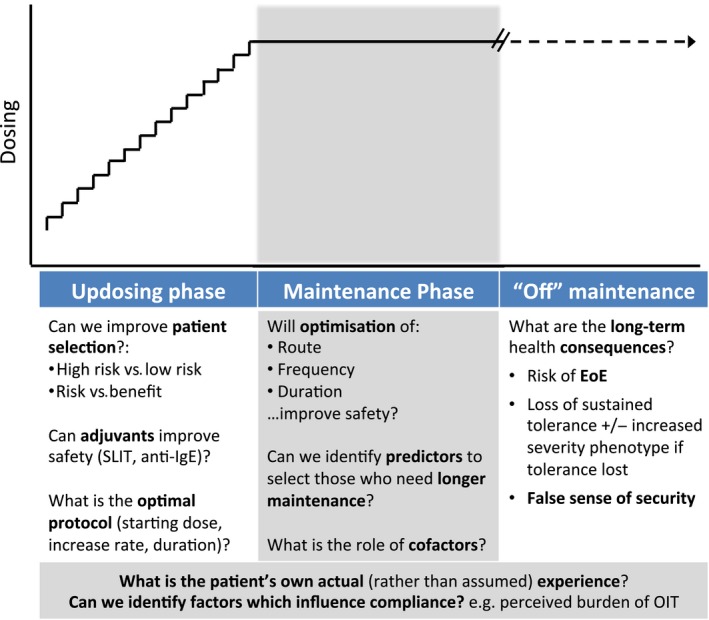
Current knowledge gaps which need to be evaluated for their impact on safety of OIT. SLIT, sublingual immunotherapy; EoE, eosinophilic oesophagitis.

Disease‐modifying treatments for food allergy are under development and have shown promising results to date, in terms of efficacy. OIT does induce desensitization, but its ability to induce permanent tolerance once ongoing exposure has stopped is limited. More research is needed into strategies – such as combining routes of exposure or identifying biomarkers of safer and successful OIT – to improve both efficacy and safety of immunotherapy treatments for food allergy (Fig. 2). Safety is concerning: most patients experience reactions to OIT doses, and severe reactions have been reported. It is for this reason that the general consensus is that OIT is not ready for clinical practice 35. This will not be resolved with the current heterogeneity in reporting adverse events. Most importantly, it is time to establish an international consensus on safety data reporting from trials of immunotherapy for food allergy.
